# Synergistic exacerbation of oral mucositis caused by IL-23 deficiency and oral *Candida albicans* exposure

**DOI:** 10.1128/mbio.01992-25

**Published:** 2025-08-27

**Authors:** John T. Dillon, Maura T. Hickey, Jessica Saul-McBeth, Trevor Glanz, Yusuf Daboul, Yashwanth R. Byreddy, Raymond Hohl, Jacqueline M. Kratch, Jia A. Thakker, Sarah L. Gaffen, Jacob Gibbs, Heather R. Conti

**Affiliations:** 1 Department of Molecular, Cellular and Developmental Biology, University of Toledo 7923, Toledo, Ohio, USA; 2 Department of Medicine, Division of Rheumatology and Clinical Immunology, University of Pittsburgh, Pittsburgh, Pennsylvania, USA; 3 Department of Radiation Oncology, The University of Toledo College of Medicine and Life Sciences 89021, Toledo, Ohio, USA; Instituto Carlos Chagas, Curitiba, Brazil

**Keywords:** oral mucositis, irradiation, head-neck irradiation, interleukin-23, candidiasis

## Abstract

Targeted head and neck irradiation (HNI) used for cancer therapy causes mucosal damage and immune dysregulation, leaving cancer patients highly susceptible to infections of the oral mucosa. Oropharyngeal candidiasis (OPC) is an opportunistic infection caused by *Candida albicans*, a commensal fungus found in up to 80% of the population at any given time. High colonization levels of *C. albicans* are known to worsen damage caused by HNI. The interleukin-23 (IL-23)–T-helper 17 (Th17) axis is a central and non-redundant mediator of immunity to OPC, and anti-cytokine biologics targeting IL-23 have come into widespread clinical use for treating various autoimmune conditions. Here, we sought to understand the consequences of IL-23 deficiency in the setting of HNI, taking advantage of a mouse model of radiation-induced oral mucositis (OM) which we combined with fungal infection. Surprisingly, mice lacking IL-23 did not show increased signs of injury to the oral mucosa when subjected to HNI. However, in mice subjected to HNI and exposed to oral *C. albicans*, damage to the oral mucosa was markedly exacerbated, accompanied by substantially increased fungal susceptibility when IL-23 was absent. Thus, IL-23-driven control of fungal infections is needed to mitigate susceptibility to OM in the high proportion of individuals who carry *C. albicans* in the mouth.

**IMPORTANCE:**

IL-23 plays key roles in expanding the T-helper 17 (Th17) cell subset during differentiation and promoting expression of cytokines, including the subset-defining cytokine IL-17 (IL-17A). While the effector cytokines produced by Th17 cells are required for host defense against extracellular microbes, especially *Candida albicans*, these can also be pathogenic during inflammatory and autoimmune disorders including psoriasis, psoriatic arthritis, and ankylosing spondylitis, among others. While IL-23 and IL-17 are similarly required for protection against oropharyngeal candidiasis (OPC), they can exert divergent functions in other forms of immune-mediated inflammation; for example, anti-IL-17 blockade or *Il17ra* gene deficiency is linked to inflammatory bowel disease, whereas loss of IL-23 is protective in this setting. We previously showed that head and neck irradiation (HNI) induces IL-17 expression in oral tissue and that IL-17 receptor (IL-17R) signaling is required for resistance against mucosal damage and OPC. In contrast, we show here that loss of IL-23 does not affect oral mucosal injury caused by HNI. However, lack of IL-23 magnifies HNI-induced susceptibility to OPC due to insufficient levels of antimicrobial peptides. Clinically, these findings suggest that patients receiving treatments that target IL-23 may not need to discontinue therapy should they require HNI, but that screening for oral *C. albicans* may be useful to help limit the risk of developing severe OM and its attendant adverse events.

## INTRODUCTION

Head and neck cancers are the sixth most common cancer type globally, and oral squamous cell carcinoma results in approximately 400,000 new diagnoses and 200,000 deaths each year (1). Head and neck irradiation (HNI), with or without combined chemotherapeutics, is the primary treatment option for these cancers. The main off-target effect of HNI is the development of oral mucositis (OM), characterized by erythematous and ulcerative lesions of the oral mucosa due to direct damage of the epithelial lining (1, 2). OM occurs in 80%–100% of HNI-treated patients (2). In addition to causing direct tissue damage resulting in loss of mucosal barrier function, radiation perturbs the immune response, contributing to increased susceptibility to oral diseases such as oropharyngeal candidiasis (OPC), herpes simplex virus infection, gingivitis, and periodontitis (3–5). Importantly, OM symptoms are more severe when these other diseases are present (2, 6). Debilitating OM symptoms can necessitate hospitalization, leading to higher medical care costs. OM is commonly treated with opioids, even though these drugs are often ineffective for oral cancer pain (2, 7). The resulting increase in opioid dosing and usage leads to various side effects (8, 9). Ultimately, these complications substantially impact quality of life and cancer progression due to altered radiation dosing schedules (2, 10–13). To date, there are no U.S. Food and Drug Administration-approved therapies that address the underlying immunopathology of OM (9).

*Candida albicans* (*C.a*) is a commensal fungus of the human microbiota colonizing up to 80% of the population at any given time (14). OPC occurs in up to 50% of patients receiving HNI, most caused by *C.a* (15, 16) Radiation damage to the oral mucosa increases the risk of bloodstream fungal infections, representing a large at-risk population with high mortality rates (4). The development of OM and the subsequent opportunistic infections are serious side effects of HNI, even leading to discontinuation of treatment at the risk of cancer progression (8, 17).

The immune response to OPC relies on the IL-23/Th17/IL-17 signaling axis. Fungal control requires IL-17R-mediated induction of neutrophil chemokines and growth factors that lead to a protective neutrophil influx to the oral tissue during OPC. IL-17R signaling in oral epithelial cells also culminates in production of antimicrobial peptides that can directly kill *C.a* (18, 19). Mice or humans deficient in *IL-23a* or genes in the IL-17 signal transduction pathway are susceptible to oral *Candida* infections (15, 18, 20–24). While IL-23 and IL-17 play highly similar protective roles during OPC, the relationship between these cytokines during other inflammatory responses is not always so congruent (25–31). For example, in inflammatory bowel disease (IBD), IL-17 is gut-protective in both mice and humans, and thus clinical trials of anti-IL-17 biologics failed or were halted early due to exacerbation of the symptoms (32, 33). In contrast, IL-23 blockade for IBD is clinically beneficial (28, 30, 34–37). Consequently, since these cytokines do not always contribute similarly to disease pathogenesis, we sought to examine IL-23 and IL-17 individually in other mucosal disease contexts.

In the setting of inflammation associated with oral mucositis (OM) caused by HNI, we have shown increased expression of both IL-23 and IL-17 in murine tongue tissue and that IL-17 signaling prevents excessive mucosal tissue damage regardless of whether there is overgrowth of *C.a* in the oral cavity (38, 39). In contrast, here, we show that lack of IL-23 in mice does not influence the inflammatory status of the oral mucosa following HNI. However, when OPC was introduced, IL-23 was required for effective antifungal responses related to HNI damage. These findings have implications for treatment of head and neck cancers and reveal unexpectedly differential roles of IL-17 and IL-23 in the oral mucosa.

## MATERIALS AND METHODS

### Mice

Mice were acquired by material transfer agreements with Genentech, Inc. (*Il23a*^−/−^; C57Bl/6J), J. K. Kolls at Tulane University (*Il22ra^−/^*^−^; C57Bl/6J), and Amgen, Inc. (*Il17ra^−/^*^−^; C57Bl/6J) (40–42). All experiments used male and female, age-matched littermate controls or wild-type (WT) controls from Jax, Inc. (antibody depletion experiments; Fig. S2). All mice were housed with food and water *ad libitum* under a 12 h dark/light cycle in a specific pathogen-free facility at the University of Toledo.

### Radiation-induced OM

Mice were subjected to HNI as described (38). Anesthetized mice were immobilized and aligned in the radiation field under a linear accelerator to deliver 22.5–24 Gy using a 6 MeV electron beam at the rate of 1,000 cGy/min in a single fraction targeted to the head and neck region. Following HNI, animals were housed in a climate and light/dark-controlled environment with free access to food and water. Animals were monitored daily for changes in weight and activity.

### *C.a* and murine OPC

*C.a* SC5314 was cultured in YPD broth the night prior to infection (43). OPC was induced 12–15 h later following HNI by sublingual inoculation with a pre-weighed cotton ball saturated in *C.a* or phosphate-buffered saline (PBS) for 75 min under anesthesia as described (44). On days 3–4 (D3–4) post-infection, fungal burden analysis of murine tongue tissue was assessed with homogenization on a GentleMACS Dissociator (Miltenyi Biotec, Auburn, CA, USA) and plating on YPD agar for CFU enumeration.

### Macroscopic and histopathologic examination

Tongues were rinsed with PBS and stained with 1% toluidine blue (Sigma-Aldrich, St. Louis, MO, USA) for 2 min, followed by a 30 s acetic acid wash to reveal ulcerative lesions as described (38). The percentage of toluidine blue-positive areas was calculated using ImageJ, and the % damage was quantified by the area of toluidine blue-positive area/surface area of the tongue. The staining on the ventral side of the tongue was not included as this included the excision site. Tissues were formalin-fixed, paraffin-embedded, and sectioned at a thickness of 5 µm. Ulcer size, mucosal thickness, and cellular infiltrate were quantified in hematoxylin and eosin (H&E)-stained tissue using an EVOS FLc microscope (Thermo Fisher Scientific, Inc., Waltham, MA, USA). Evaluators blinded to the treatment groups analyzed the tissue sections.

### Antibody administration

Anti-IL-23p19 (BE0313, BioXcell, Lebanon, NH, USA) neutralizing Ab was administered at 100 µg/kg intraperitoneally on D7 post-irradiation. Isotype control InVivoMab rat IgG1 anti-horseradish peroxidase (BE0088, BioXcell, Lebanon, NH, USA) was administered to WT mice.

### Immunohistochemistry

Tissue sections were dehydrated with xylene and ethanol gradient, and antigen retrieval and blocking were performed. BioLegend (San Diego, California, USA) Ultra Streptavidin HRP Kit (Multi-Species, DAB) was followed using manufacturer’s protocol. Sections were labeled with antibodies myeloperoxidase (MPO, IL-1α, IL-6, Ki67, and S100a8 (R&D Systems, Minneapolis, MN, USA) or β-defensin 3 (Santa Cruz Biotechnology, Dallas, TX, USA) with secondary HRP-conjugated antibody. Tissue was counter-stained with hematoxylin. Sections were imaged on EVOS FLc microscope (Thermo Fisher Scientific, Inc., Waltham, MA, USA). For quantification of percent area positive staining, images of the entire tissue were taken, and using ImageJ (45), staining intensity was measured within the suprabasal epithelial layer (SEL) and basal epithelial layer (BEL). Evaluators were blinded to sample identity.

### Real-time PCR

Total RNA was extracted with TRI reagent (Sigma-Aldrich, St. Louis, MO, USA) and RNA (1 µg) reverse-transcribed by Lunascript RT Supermix (New England Biolabs, Ipswitch, MA, USA). Quantitative PCR was performed using Luna Universal qPCR Master Mix (New England Biolabs, Ipswich, MA, USA) and a Quant Studio 3 detection system (Applied Biosystems, Waltham, MA, USA), normalized to *Gapdh*. Primers were PrimeTime qPCR Primer Assays (IDT Integrated DNA Technologies, Coralville, IA, USA). Assays were performed in biological triplicate in technical triplicate.

### Statistics

At least three biological replicates were included per group. Experiments were repeated at least two times, specified in the figure legends. Normally distributed data were analyzed via analysis of variance (ANOVA) with Tukey’s *post hoc* analysis or Student’s *t*-test. Non-parametric data were analyzed by Kruskal–Wallis or Mann–Whitney using GraphPad Prism (V8.4.3). *P*-values < 0.05 were considered significant.

## RESULTS

### The contribution of IL-23 is dispensable during HNI-induced OM

**Fig 1:**
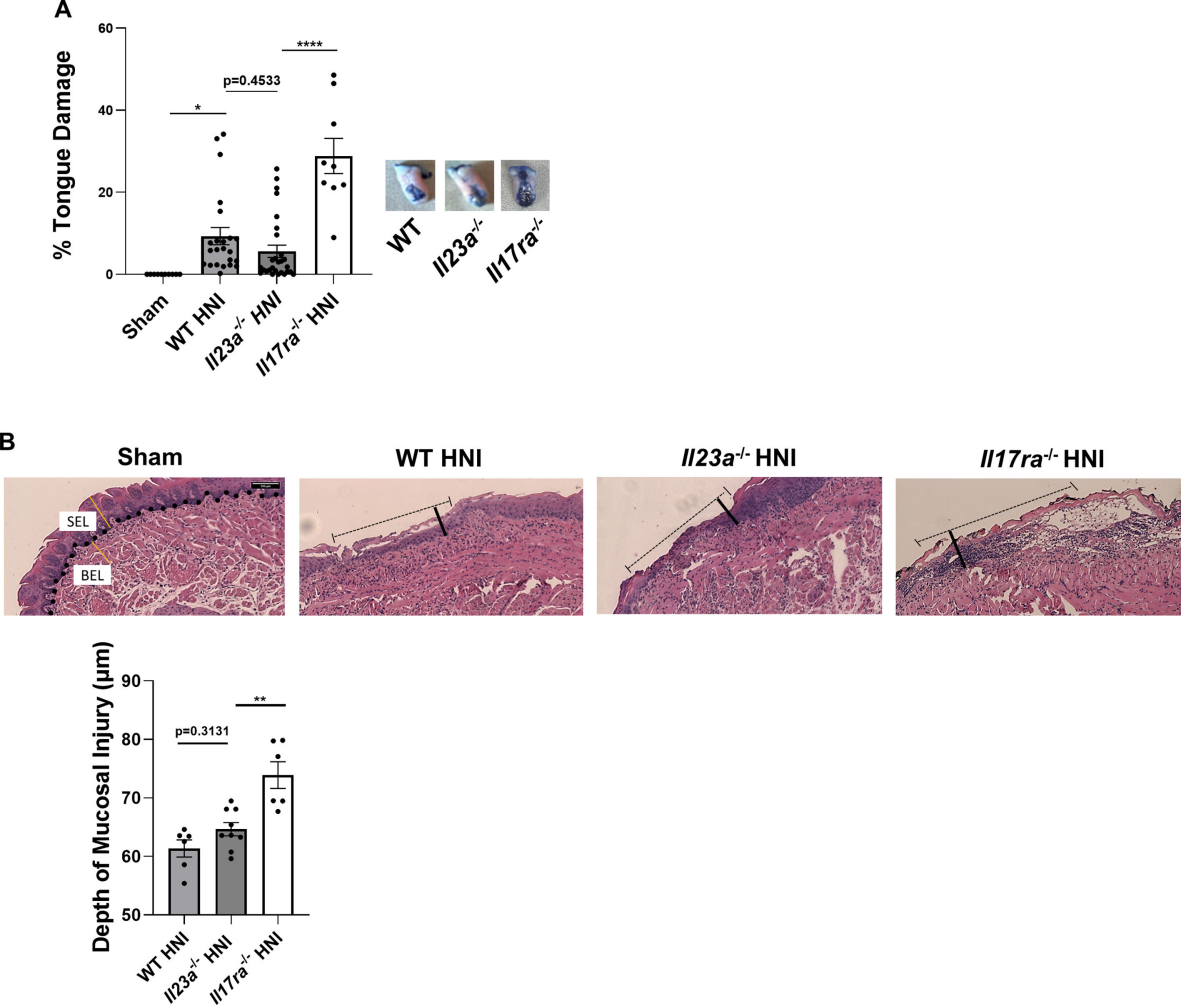
The contribution of IL-23 is dispensable during HNI-induced OM**.** (**A**) Quantification of toluidine blue positive staining area by determining the surface area of the tongue positive for blue staining compared to the total surface area of tongue on each mouse 11 days post-head and neck radiation. The staining on the ventral side of the tongue was not included in the quantification since this included the excision site. Data pooled from at least three experiments, *n* = minimum three mice per cohort per experiment. Analyzed by ANOVA with Tukey’s *post hoc*. (**B**) Representative H&E staining of tongue tissue on D11 post-HNI, images taken at 10× magnification. Basal stem cell layer (represented by dotted line), Ulcer length (represented by dashed lines), SEL, and BEL denoted by yellow lines. Quantification of mucosal tissue injury depth within the SEL was performed in ImageJ and normalized to sham, *n* = at least three mice per group. Data represents at least three pooled experiments analyzed by ANOVA with Tukey’s *post hoc* (*P < 0.05, **P < 0.01, ****P < 0.0001).

To determine if the increase in IL-23 expression is protective during OM (38), *Il23a*^−/−^ mice were subjected to 22.5 Gy of radiation targeted to the head and neck region. *Il17ra*^−/−^ mice were included as a control since we previously showed they exhibit elevated OM damage upon HNI (38). Tongue damage was quantified 11 days post-irradiation by toluidine blue staining, which marks areas of mucosal barrier disruption. As shown, ulcerative lesions of similar sizes were detected in *Il23a*^−/−^ mice (9% of total tongue) and WT mice (6%), and both cohorts exhibited reduced damage compared to *Il17ra*^−/−^ mice (29%) (Fig. 1A). Similar to *Il23a*^−/−^ mice, there was no difference in damage outcomes after α-IL-23a blockade or isotype control treatment compared to WT (Fig. S1).

To better understand the extent of damage to oral epithelium during OM, the tongue was evaluated histologically for damage parameters. Consistent with the toluidine blue staining, *Il23a*^−/−^ and WT mice showed similar tissue injury depths into the SEL following HNI, which were higher than sham-irradiated mice and lower than *Il17ra*^−/−^ mice (Fig. 1B). These findings show that loss of IL-23 does not incur OM susceptibility following HNI compared to IL-23 sufficiency and differs from damage seen in the absence of IL-17RA.

### HNI-induced neutrophil recruitment and cytokine expression do not require IL-23

**Fig 2:**
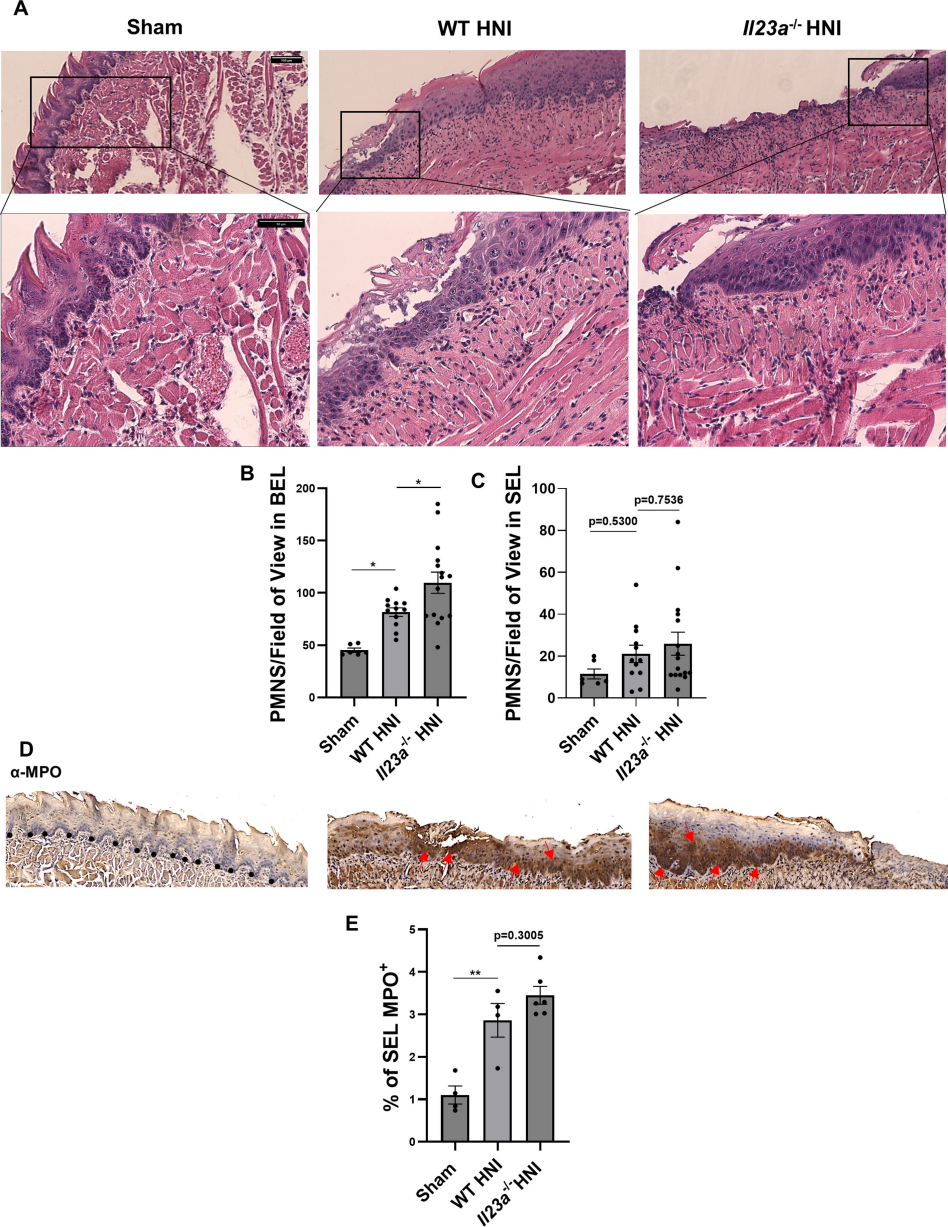
Neutrophil recruitment to the tongue tissue is unaffected by the lack of IL-23 following HNI. (**A**) Staining by H&E of sectioned tongues on D11 post-HNI, images taken at 10× magnification (scale bar 100 µm) followed by 20× zoom in (scale bar 50 µm) of selected areas. Data represent two experimental repeats, *n* = minimum 3 mice per group. (**B, C**) Quantification of neutrophils in the SEL and BEL on D11 post-HNI. Quantification analyzed by ANOVA with Tukey’s *post hoc*. Evaluators blinded to the treatment group and mouse cohort analyzed the tissue sections. (**D**) Representative α-MPO-staining of HNI-treated and untreated tongue tissue counterstained with hematoxylin, images taken at 10× magnification. Dotted line indicated the basal stem cell layer. Data represent two experimental repeats, *n* = minimum 3 mice per group. Arrows represent positive MPO staining. (**E**) Quantification of % area positive for staining in the SEL across entire tissue was analyzed in ImageJ. Analyzed by ANOVA with Tukey’s *post hoc*. Data shown as means ± SEM (*P < 0.05, **P < 0.01).

During early stages of OM, the inflammatory response is amplified by production of reactive oxygen species, followed by amplified induction of cytokines such as IL-1 and IL-6 (2). The actions of these inflammatory mediators lead to an influx of MPO-producing macrophages and neutrophils, which further augment mucosal tissue damage (2, 46–48). Peak damage levels during OM coincide with the upregulation of IL-17, which we showed to be required for epithelial repair and a balanced neutrophil response (47). In *Il17ra*^−/−^ mice, the excess accumulation of neutrophils was due to the dysregulation of other cytokines, for example, IL-1α (38). Therefore, we interrogated how the absence of IL-23 affected the polymorphonuclear cell response when OM damage was maximal (D11 post-irradiation) (47, 48). The numbers of neutrophils in the BEL were modestly higher in *Il23a*^−/−^ mice (Fig. 2A and B). However, in the SEL, where most of the overt damage occurs, neutrophil levels were similar (Fig. 2A and C). Levels of MPO in the tongue are a proxy measure of the severity of ulceration caused by radiation (2, 46, 49–51). Immunohistochemical analysis showed similar levels of MPO in *Il23a*^−/−^ compared to WT mice (Fig. 2D and E), suggesting that neutrophils in these mice were equivalently functionally competent.

**Fig 3:**
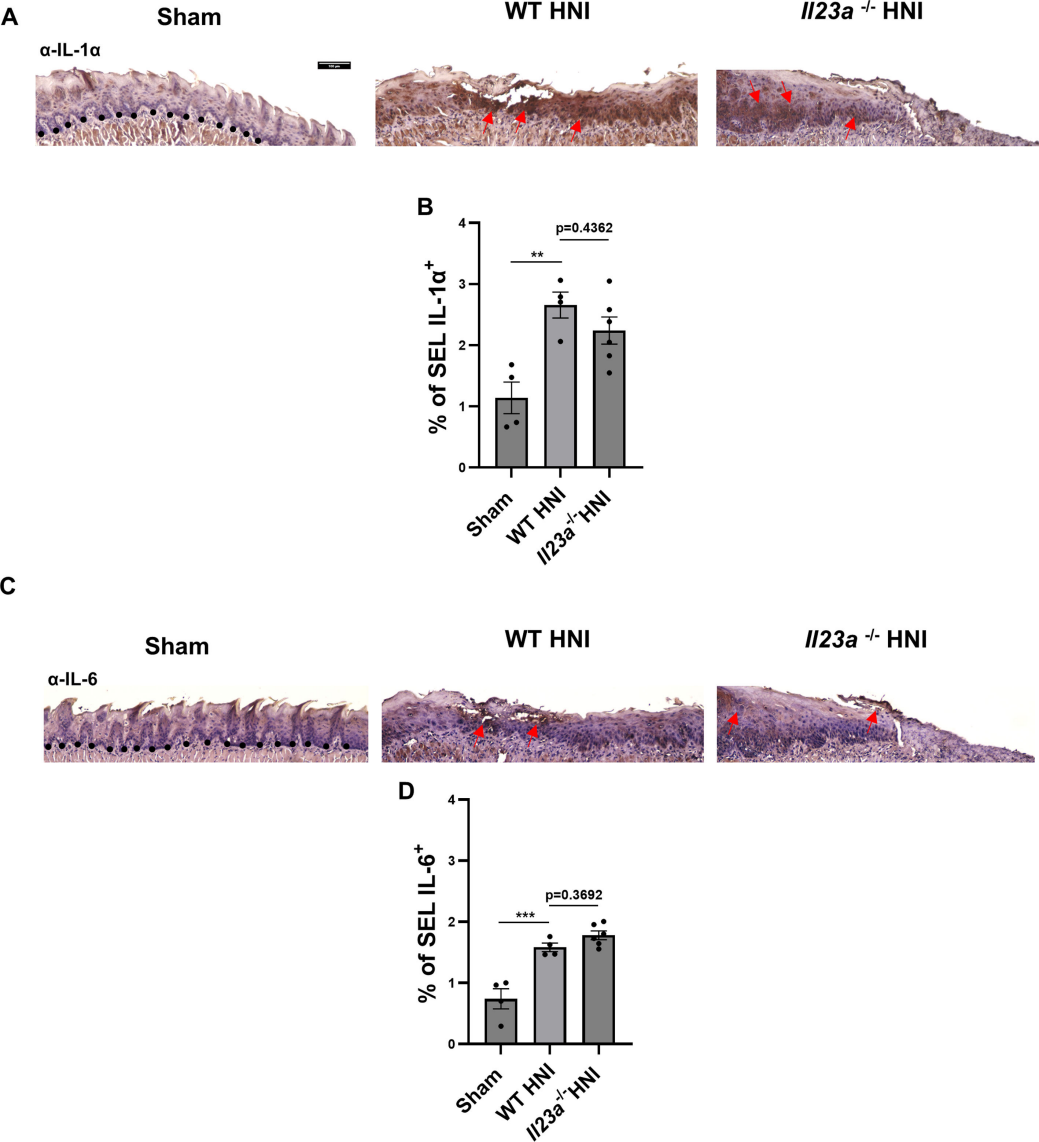
Absence of IL-23 does not alter expression of other proinflammatory cytokines following HNI. (**A**) Representative α-IL-1α staining of HNI-treated and untreated tongue tissue counterstained with hematoxylin, images taken at 10× magnification. Arrows represent positive staining. (**B**) Quantification of % area positive for IL-1α staining in the SEL across entire tissue was analyzed in ImageJ. Analyzed by ANOVA with Tukey’s *post hoc*. Data represent two experimental repeats, *n* = at least three mice per group. Data shown as means ± SEM. (**C**) Representative α-IL-6 staining of HNI-treated and untreated tongue tissue counterstained with hematoxylin, images taken at 10× magnification. Arrows represent positive staining. (**D**) Quantification of % area positive for IL-6 staining in the SEL across entire tissue was analyzed in ImageJ. Analyzed by ANOVA with Tukey’s *post hoc*. Data represent two experimental repeats, *n* = at least three mice per group. Data shown as means ± SEM (**P < 0.01, ***P < 0.001).

Given the modest increase in the quantity of neutrophils recruited to the BEL in *Il23a*^−/−^ mice, we next determined if dysregulation of other inflammatory mediators accounted for this elevation. IL-1α (Fig. 3A and B) and IL-6 protein levels were similar between WT and *Il23a*^−/−^ mice following HNI and higher than sham-irradiated tongues (Fig. 3C and D). *Il17a* transcript levels were reduced in *Il23a*^−/−^ mice, with no apparent compensatory induction of other Th17 cytokines such as *Il22* in mice lacking IL-23 (Fig. 2A and B). Moreover, radiation exposure did not induce *Il22* above sham levels in WT mice during peak damage on D11 (Fig. 2B). Furthermore, mice lacking the IL-22 receptor (*Il22ra1^−/−^*) were not more susceptible to OM than WT mice, indicating the contribution of IL-22 post-irradiation was negligible (Supp Fig. 1C), unlike IL-17. Overall, these findings suggest that the absence of IL-23 does not exacerbate the inflammatory response post-HNI, which presents a marked contrast to the impact of IL-17R signaling (38).

### Oral epithelia of *Il23a*^−/−^ mice have an enhanced proliferative capacity

**Fig 4:**
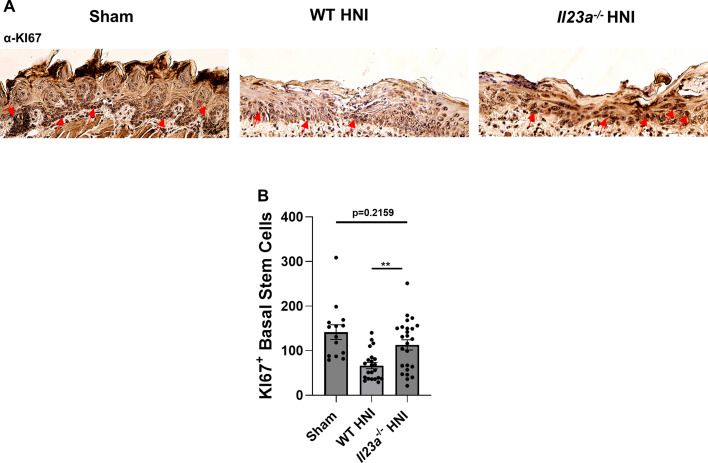
Oral epithelia of *Il23a*^−/−^ mice have an enhanced proliferative capacity. (**A**) Representative images of proliferation marker Ki67 staining of HNI-treated and untreated tongue tissue, images taken within ulcer region in the dorsal portion of the tongue. Data represent two experimental repeats, *n* = minimum three mice per group per experiment. Images taken at 20× magnification. Arrows represent positive Ki67 staining. (**B**) Quantification of KI67^+^ cells within the basal stem cell layer of the tongue obtained on D11 following HNI, evaluators blinded to the treatment group and mouse cohort analyzed the tissue sections. Analyzed by ANOVA with Tukey’s *post hoc*. Data shown as mean + SEM (**P < 0.01).

Oral keratinocyte proliferation and differentiation is critical for mucosal repair during OM resolution following discontinuation of radiation exposure (12, 38, 52, 53). Intraoral wound repair is a complex process that is notably different from cutaneous skin and requires distinct transcriptional control of inflammatory and repair mechanisms throughout the types of oral tissue (54–56). To understand if IL-23 plays a role in oral mucosal regeneration in this setting, Ki67 levels were assessed to mark actively proliferating cells within tongue. WT mice subjected to HNI showed decreased levels of Ki67^+^ cells compared to *Il23a*^−/−^ mice within the damaged mucosae, while the number of proliferating cells in the *Il23a*^−/−^ mice was comparable to sham-irradiated mice (Fig. 4A and B). Together, these results suggest that reepithelization may occur more rapidly in *Il23a*^−/−^ mice, potentially accounting for the modestly lower levels of damage compared to IL-23-sufficient mice (Fig. 1).

### *Il23a*^−/−^ mice have increased mucosal damage and fungal susceptibility after HNI

**Fig 5:**
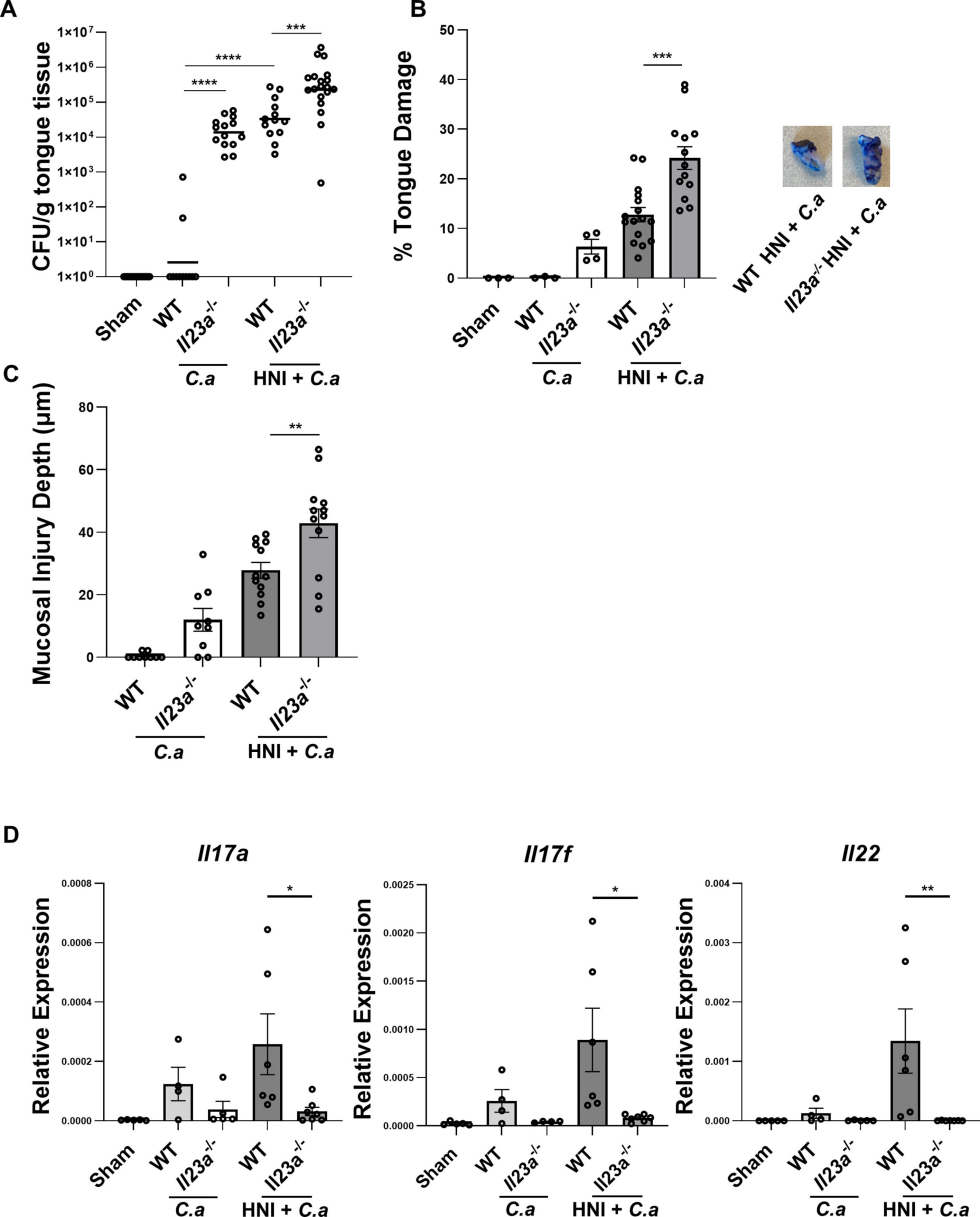
*Il23a*^−/−^ mice have increased mucosal damage and fungal susceptibility after HNI. (**A**) WT and *Il23a*^−/−^ mice were subject to HNI and 16 h later infected sublingually with *C.a*. Tissue was harvested on D3–4 post-infection, and CFU/g of tongue tissue was assessed between WT and *Il23a*^−/−^ mice treated with OPC or HNI + *C.a*. Data pooled from at least three experiments, *n* = minimum three mice per cohort per experiment. Analyzed by Mann–Whitney *U* test. Data shown as mean + SEM. (**B**) D3-4 quantification of toluidine blue staining by determining the surface area of the tongue positive for blue staining compared to total surface area for each mouse. Data pooled from at least three experiments, *n* = minimum three mice per cohort per experiment. Analyzed by one-way ANOVA with Tukey’s *post hoc*. (**C**) Quantification of mucosal tissue injury depth within the SEL was performed in ImageJ and normalized to sham. Data represent at least three pooled experiments, *n* = at least three mice per group. Analyzed by one-way ANOVA with Tukey’s *post hoc*. Evaluators blinded to the treatment group and mouse cohort analyzed the tissue sections. (**D**) Expression differences relative to GAPDH of Th17 effector cytokines *Il17a*, *Il17f*, and *Il22* on D4 post- *C.a-*only or HNI + *C.a*. Data include two experimental repeats, *n* = minimum two to three mice per group per experiment. Analyzed by one-way ANOVA with Tukey’s *post hoc* (*P < 0.05, **P < 0.01, ***P < 0.001, ****P < 0.0001).

IL-17 is protective during the anti-fungal immune response to *C.a* following immunosuppressive irradiation by mitigating oral mucosal tissue damage and fungal susceptibility (39). Mice, unlike humans, do not carry *C.a* as a commensal microbe and are hence immunologically naïve to this organism (57, 58). However, *C.a* is present in up to 80% of healthy humans and thus has the potential to influence IL-17-driven immune responses. Accordingly, to elucidate the role of IL-23 during OPC infection post-HNI, WT and *Il23a*^−/−^ mice were subjected to HNI and infected orally with a virulent strain of *C.a* (SC5314). Tongue fungal burdens were assessed at a time point at which healthy WT mice are known to clear infection (3–4 days) (19). WT mice exposed to *C.a* cleared the infection by D4. As expected, *Il23a*^−/−^ mice maintained high fungal burdens at the same time point (Fig. 5A) (18). However, WT mice exposed to HNI + *C.a* were even more susceptible to infection, indicated by persistent presence of fungal burden on D4. Furthermore, *Il23a*^−/−^ mice treated with HNI *+C.a* had even higher fungal burdens compared to WT HNI + *C.a-*treated mice or *Il23a*^−/−^ *C.a*-only controls (Fig. 5A). These data demonstrate that HNI increases fungal susceptibility and that IL-23 is required for antifungal immunity post-HNI.

Next, mucosal tissue damage was assessed during OPC and when mice were exposed to HNI + *C.a*. While *Il23a*^−/−^ showed increased damage compared to WT mice when infected with *C.a* without HNI, there was markedly more oral mucosal tissue damage in the condition of HNI + *C.a* in IL-23-deficient mice compared to WT controls (Fig. 5B). The increased surface area of damage across the tongue tissue correlated with the depth of mucosal tissue injury in regions of localized damage (Fig. 5C). These data show that *Il23a*^−/−^ mice experience a more severe loss of mucosal integrity compared to WT mice and that IL-23 is required for protection after HNI when there is an overgrowth of *C.a*.

We next determined whether the absence of IL-23 led to IL-17 deficiency in the scenario of radiation damage and infection, as is the case for each condition individually. Indeed, expression of Th17 effector cytokines (*Il17a*, *Il17f*, and *Il22*) was more highly elevated when WT mice received HNI + *C.a* compared to *C.a*-only and was all reduced in *Il23a*^−/−^ mice (Fig. 5D).

### IL-23 deficiency impacts antimicrobial peptides but not neutrophil responses or inflammatory cytokines after HNI

**Fig 6:**
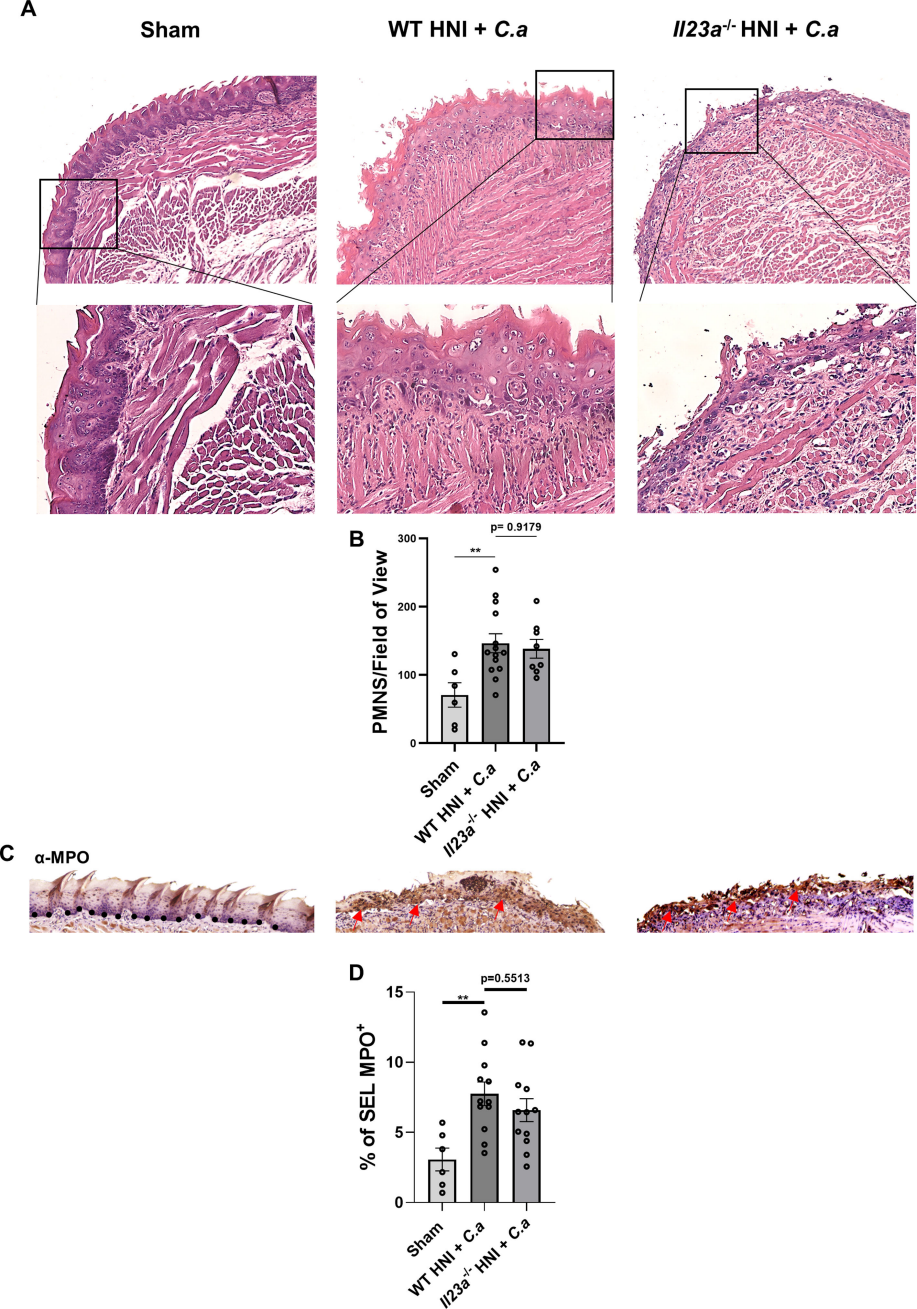
The absence of IL-23 does not lead to aberrant neutrophil responses after irradiation. (**A**) Staining by H&E of sectioned tongues on D4 post-HNI + *C.a*, images taken at 10× magnification (scale bar 100 µm) followed by 20× zoom in (scale bar 50 µm) of selected areas. Data represent three experimental repeats, *n* = minimum three mice per group. (**B**) Quantification of neutrophils on D4 post-HNI + *C.a*. Quantification analyzed by ANOVA with Tukey’s *post hoc*. Evaluators blinded to the treatment group and mouse cohort analyzed the tissue sections. (**C**) Representative α-MPO-staining of HNI + *C.a*-treated and *C.a*-untreated tongue tissue on D4 counterstained with hematoxylin, images taken at 10× magnification. (**D**) Quantification of % area positive for MPO staining in the SEL across entire tissue was analyzed in ImageJ. Analyzed by ANOVA with Tukey’s *post hoc*. Data shown as means ± SEM (**P < 0.01).

Inflammatory mediators involved in the neutrophil response are dysregulated in *Il17ra^−^*^/−^ mice post-irradiation, contributing to increased oral mucosal damage and fungal susceptibility (39). Even though *Il23a*^−/−^ mice failed to induce *Il17a*, there were similar neutrophil numbers in the oral mucosa following HNI + *C.a* compared to WT (Fig. 6A and B). Additionally, activation of neutrophils in WT and *Il23a*^−/−^ tongues exposed to HNI + *C*.a was similar, with comparable MPO activity across the tissue (Fig. 6C and D). Thus, the increased fungal burden after HNI in mice lacking IL-23 cannot be explained by neutrophil defects in either population size or activity.

**Fig 7:**
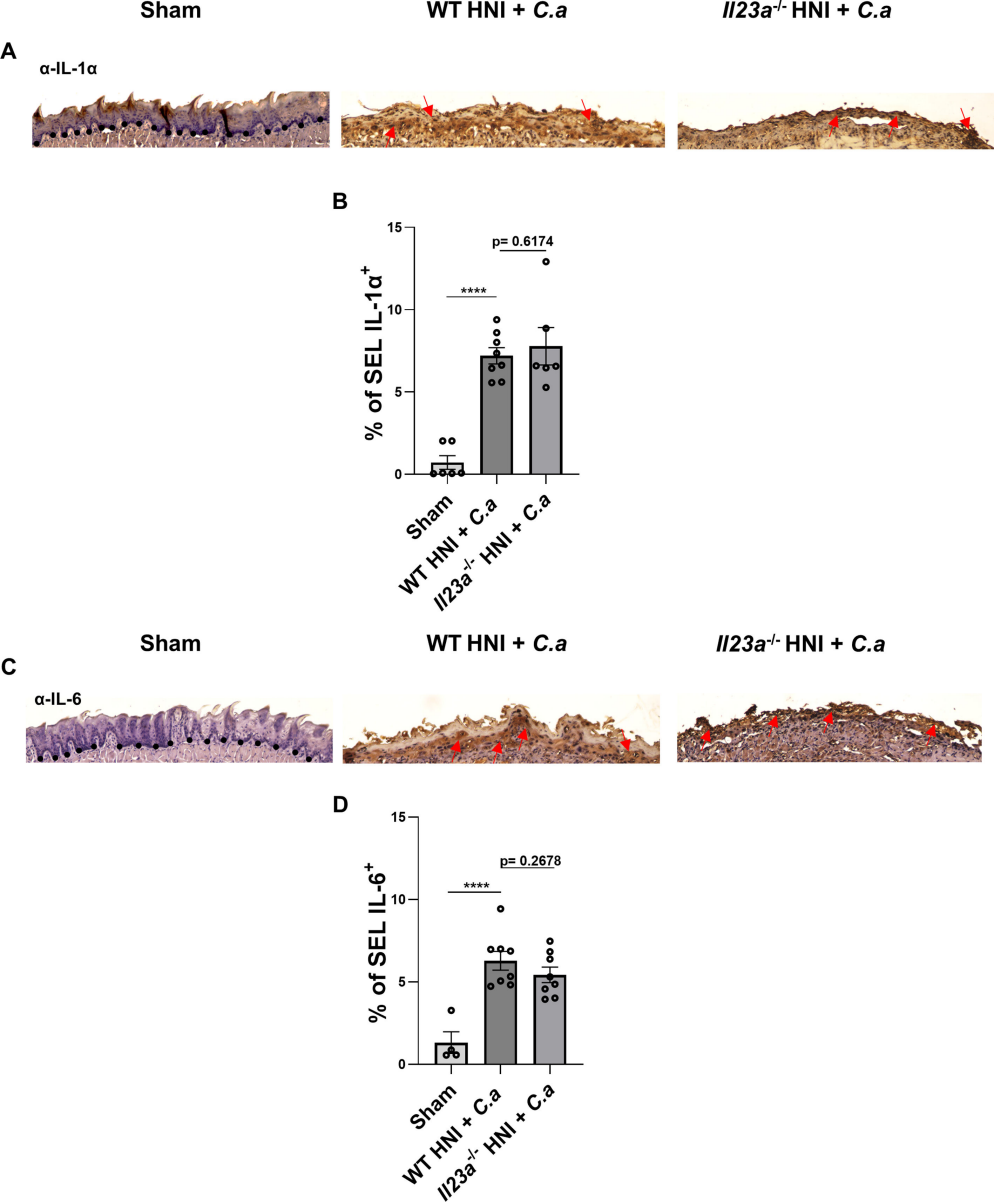
The absence of IL-23 does not alter proinflammatory mediators during OPC post-HNI. (**A**) Representative α-IL-1α-staining of HNI + *C.a*-treated and *C.a*-untreated tongue tissue on D4 counterstained with hematoxylin, images taken at 10× magnification. Arrows represent positive staining. (**B**) Quantification of % area positive for IL-1α staining in the SEL across entire tissue was analyzed in ImageJ. Analyzed by ANOVA with Tukey’s *post hoc*. Data represent two experimental repeats, *n* = at least three mice per group. Data shown as means ± SEM. (**C**) Representative α-IL-6 staining of HNI + *C.a*-treated and *C.a*-untreated tongue tissue on D4 counterstained with hematoxylin, images taken at 10× magnification. Arrows represent positive staining. (**D**) Quantification of % area positive for IL-6 staining in the SEL across entire tissue was analyzed in ImageJ. Analyzed by ANOVA with Tukey’s *post hoc*. Data represent two experimental repeats, *n* = at least three mice per group. Data shown as means ± SEM (****P < 0.0001).

Since *Il23a*^−/−^ mice were more susceptible to fungal infection after HNI yet exhibited no apparent neutrophil defect, we next asked whether inflammatory cytokines other than IL-17 might account for the increased damage and exacerbated susceptibility to OPC. While IL-1α accounts for the excess damage in irradiated *Il17ra*^−/−^ mice seen previously (38), both the SEL and BEL regions of the damaged tongue tissue of *Il23a*^−/−^ HNI + *C.a-*treated mice showed similar expression of IL-1α or IL-6 compared to irradiated and infected WT mice (Fig. 7A through D).

**Fig 8:**
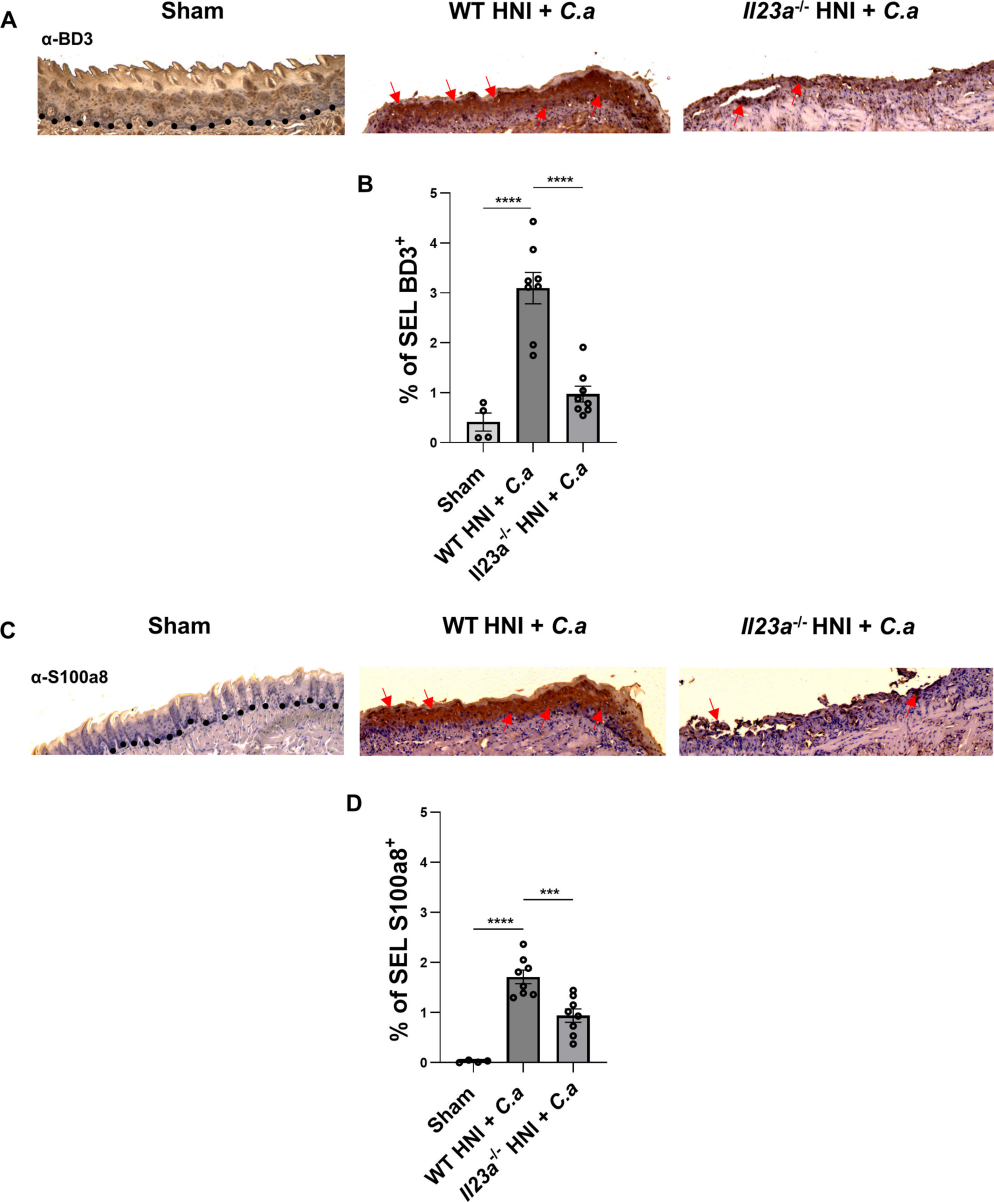
IL-23 is required for effective antimicrobial peptide responses during OPC associated with HNI. (**A**) Representative α-BD3-staining of HNI + *C.a*-treated and *C.a*-untreated tongue tissue on D4 counterstained with hematoxylin, images taken at 10× magnification. Arrows represent positive staining. (**B**) Quantification of % area positive for BD3 staining in the SEL across entire tissue was analyzed in ImageJ. Analyzed by ANOVA with Tukey’s *post hoc*. Data represent two experimental repeats, *n* = at least three mice per group. Data shown as means ± SEM. (**C**) Representative α-S100A8-staining of HNI + *C.a*-treated and *C.a*-untreated tongue tissue on D4 counterstained with hematoxylin, images taken at 10× magnification. Arrows represent positive staining. (**D**) Quantification of % area positive for S100a8 staining in the SEL across entire tissue was analyzed in ImageJ. Analyzed by ANOVA with Tukey’s *post hoc*. Data represent two experimental repeats, *n* = at least three mice per group. Data shown as means ± SEM (**P < 0.01, ****P < 0.0001).

IL-17 signaling potently regulates antifungal antimicrobial peptides, including β-defensins, calprotectin (S100A8/9), and small proline-rich proteins (SPRRs) (18, 19, 38, 57, 59–62) *Il23a*^−/−^ mice exposed *to* HNI *+ C.a* had considerably lower levels of β-defensin 3 (BD3) and S100A8 protein in tongue tissue compared to WT mice with HNI + *C.a* (Fig. 8A and B). These data suggest that the consequence of IL-23 deficiency is reduced antifungal antimicrobial peptide (AMP) expression and not neutrophil defects (18, 19, 63).

## DISCUSSION

OM is the most serious non-hematological complication of targeted radiotherapy for head and neck cancers, yet effective treatments are lacking (2). This is due in part to an incomplete understanding of the pathophysiology of OM. Also missing are well-defined biomarkers of OM disease progression and severity that might help guide interventions (64). A more in-depth understanding of the inflammatory processes implicated during the stages of OM is therefore warranted. Among the parameters in this regard are immune mediators that control secondary infections such as OPC that severely impact patient quality of life, even to the point of prompting treatment cessation (15, 16). Hence, investigation of the IL-23/IL-17 immune axis during radiation damage was necessary to understand if blocking this pathway may be an effective treatment option during cancer and the related secondary diseases of OM and OPC. The existing therapies for OM mitigate symptoms, rather than prevent the onset of disease (8, 9, 65). In addition to the preventative aspects of novel therapies for OM, better-designed treatment regimens should also consider how the host response to infection will be affected.

In certain disease contexts, there is not a direct line connecting the function of IL-23 and IL-17, like there is in OPC (28). This study provided another example of the dichotomy between IL-17RA signaling and IL-23 in the setting of OM during both genetic and drug-induced deficiency. Our findings suggest that with the specific absence of IL-17R signaling, there is inflammatory dysregulation leading to increased oral mucosal damage (38). Even so, targeting of IL-23 genetically or with antibody blockade does not skew immune pathways involved either in the development or progression of OM, despite *Il17a* levels being down. This informs potential therapeutic options for OM but only in the absence of secondary infections. Consequently, in this study, we also considered radiation-induced oral mucosal damage in the context of the most common infection associated with the loss of barrier function (66, 67). Understanding both clinical situations of OM with, and without, OPC is important though. Since nearly all individuals receiving HNI will develop OM (2), OPC will be present in a sizable portion of these patients (66, 67).

Our results indicate that when *C.a* overgrowth is present in the oral cavity, the antifungal contribution of IL-23 is then required. While the *Il23a*^−/−^ mice may not have had a dysregulated neutrophil response, there was still increased fungal susceptibility compared to WT mice. IL-17 is necessary not only for a balanced neutrophil response during OPC but also for production of antimicrobial peptides that are required for efficient clearance of *Candida* from the oral epithelia (18, 19, 38, 39). In addition to direct candidacidal activity, AMPs are potent immunomodulatory mediators that can stimulate production of cytokines and chemokines as well as promote wound healing (59, 68–71). Although the lack of these AMPs likely led to less direct killing of *Candida*, we presume that the involvement of these molecules in the overall immune response was limited since other inflammatory readouts were not skewed in the *Il23a*^−/−^ mice during HNI-alone or HNI + *C.a*. This aligns with previous studies showing that the IL-17RA signaling pathway terminates with expression of BD3, since this AMP shows no immunoregulatory properties of its own during OPC (59). An interesting implication of this study, then, is the therapeutic potential of BD3 and S100A8/9 for the treatment of *Candida* infections associated with excessive radiation-associated damage and inflammation. These treatment options could also extend to other forms of mucosal candidiasis, such as vulvovaginal, where clearance of *Candida* is needed without further intensifying the inflammatory response in the tissue. While we identified a role for BD3 and S100A8, the specific contributions of other AMPs during radiation-induced OM and OPC infection will need to be determined, for example, SPRRs (62). In addition, important cellular sources of β-defensins include not only the epithelial cells of the gingiva, buccal mucosa, and tongue but also the salivary glands. At the early time points of the HNI + *C.a* model, limited radiation-induced destruction of the salivary glands has developed, so the role of AMPs when there is substantial salivary damage will need to be investigated (5, 18).

Here, we provide a better molecular understanding of the oral mucosa during radiation exposure, which is required for informed therapeutic development. The broad immunoregulatory effects of potential cancer immunotherapies must be understood as they relate to the secondary development of oral diseases (72). The use of Th17 pathway-related modulators to combat autoimmune conditions is on the rise due to the efficacy of these drugs in dampening inflammation and lessening disease scores. It is reasonable to assume that a portion of the individuals receiving these treatments could develop a malignancy requiring HNI (21, 31). In such scenarios, managing the cancer, particularly head and neck squamous cell carcinomas, will necessitate coordination with the therapies for their pre-existing autoimmune conditions. Additionally, the pro-tumorigenic properties of IL-17 and IL-23 make it likely that agents blocking these cytokines would be good candidates for anti-cancer therapy (73–76). It is probable that these anti-IL-17 and anti-IL-23 cancer immunotherapies would be prescribed in combination with radiotherapy. These treatment modalities will also require careful planning and monitoring. For example, the use of risankizumab (Skyrizi) may be considered even if radiation-induced OM develops since this monoclonal antibody targets IL-23. However, therapies that target IL-17 or the IL-17R, like secukinumab (Cosentyx) or brodalumab (SILIQ), may not be indicated since OM symptoms could be exacerbated (37, 77–80). In the long term, we envision that the HNI + OPC system can be combined with oral cancer models as a platform to assess the impact of cancer therapeutics on the complex dynamics of oral tissue, especially since many pre-clinical immunotherapies are tested in combination with radiotherapy (81). We have established an ideal *in vivo* system to test these regimens as it considers the cancer treatment efficacy, along with the consequences for OM and mycobiome alterations. We speculate that this may allow insights into why certain immunotherapies for head and neck cancers have low clinical efficacy and potentially develop strategies to improve this using anti-cytokine biologics that target oral inflammatory signaling pathways (82, 83).

In conclusion, our findings further clarify the multifaceted roles of IL-23 and IL-17 in the oral mucosa following HNI and OPC. Intact IL-17R signaling mechanisms are required post-irradiation to allow tissue regeneration and to ensure proper regulation of other inflammatory cytokines like IL-1α. This leads to a balanced neutrophil response and less mucosal damage than when IL-17RA is absent. In contrast, when IL-23 was missing, various inflammatory readouts (IL-1α, IL-6, and IL-22) were not dysregulated, neutrophil levels were controlled, and the overall OM symptoms were similar to levels in WT mice. Yet, it was during radiation damage along with exposure to *C.a* that IL-23 was necessary for control of mucosal damage and fungal growth through induction of AMPs including BD3 and S100A8/9 that kill *C.a* directly. By extension then, this study suggests that a patient receiving an anti-IL-23-related biologic for an autoinflammatory condition may be able to remain on the treatment during a subsequent course of irradiation, if they continue to respond to antifungal drugs for the *C.a* infections that typically develop.

## SUPPLEMENTAL MATERIAL

Fig. S1 mbio.01992-25-s0001.tif Tongue damage in head-neck irradiation with cytokine blockade.

Fig. S2 mbio.01992-25-s0002.tif Cytokine-mediated tongue damage in head-neck irradiation.

Legends mbio.01992-25-s0003.docx Legends for Fig. S1 and S2.

ASM does not own the copyrights to Supplemental Material that may be linked to, or accessed through, an article. The authors have granted ASM a non-exclusive, world-wide license to publish the Supplemental Material files. Please contact the corresponding author directly for reuse.
