# Supplementary figures and images for: Synergistic exacerbation of oral mucositis caused by IL-23 deficiency and oral Candida albicans exposure
Source: mBio. 2025 Aug 27;16(10):e01992-25. doi: 10.1128/mbio.01992-25 (PMC12506028; doi:10.1128/mbio.01992-25)

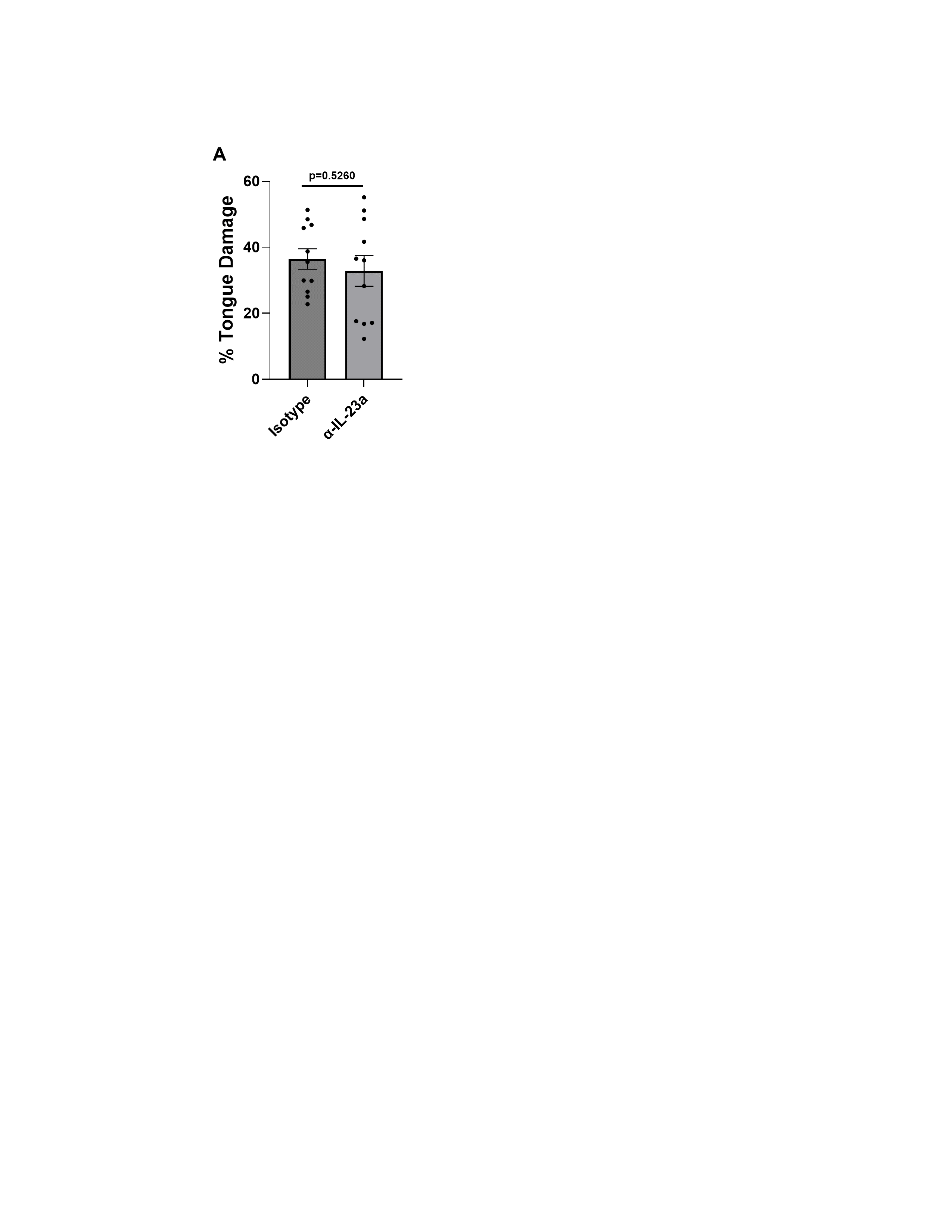

Supplement: Fig. S1 — Tongue damage in head-neck irradiation with cytokine blockade. [file mbio.01992-25-s0001.tif]

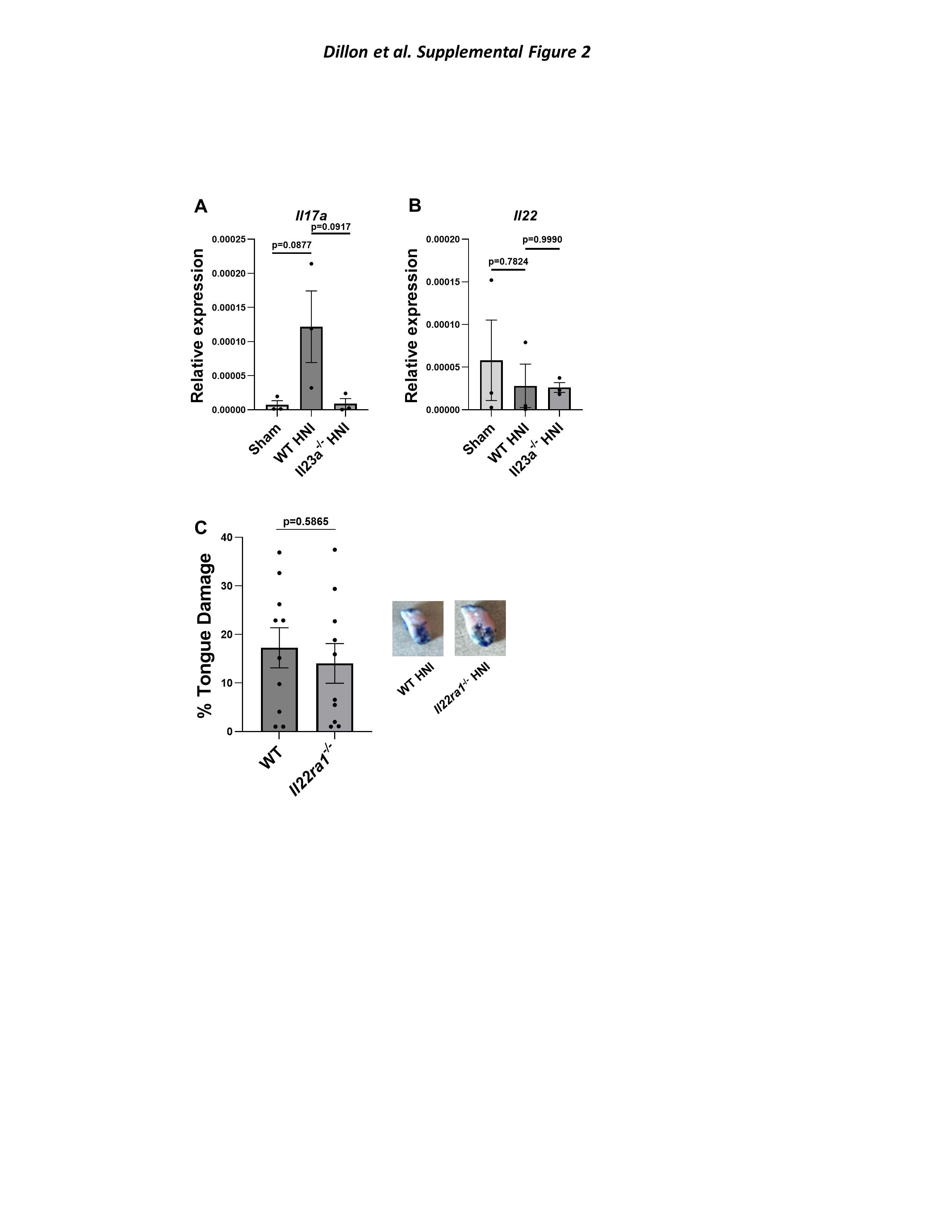

Supplement: Fig. S2 — Cytokine-mediated tongue damage in head-neck irradiation. [file mbio.01992-25-s0002.tif]
