# Supplementary material for: Synergistic exacerbation of oral mucositis caused by IL-23 deficiency and oral Candida albicans exposure
Source: mBio. 2025 Aug 27;16(10):e01992-25. doi: 10.1128/mbio.01992-25 (PMC12506028; doi:10.1128/mbio.01992-25)
Supplement: Legends — for Fig. S1 and S2. [file mbio.01992-25-s0003.docx]

**Supplemental Figure Legends**

**Supplemental Figure 1. Antibody blockade of IL-23 does not exacerbate OM Damage (A)** Mice were subject to 24-25gy radiation and treated on days 7 with α-IL-23a (100µg/Kg) or mouse IgG1 and damage quantified on day 11. n = 11 mice per group. Data combined from two independent experiments. Analyzed by Student’s t test. Data shown as means ± SEM.

**Supplemental Figure 2. Lack of IL-22 does not exacerbate damage during HNI-induced OM. (A-B)** Expression differences relative to GAPDH of *Il17a* and *Il22* on between sham, WT and *Il23a*^-/-^ mice D11 following HNI. Data represent minimum 2 experimental repeats, *n* = 3 mice per group per experiment. Analyzed by one-way ANOVA with Tukey’s post hoc. (C) Day 11 quantification of toluidine blue staining by determining the surface area of the tongue positive for blue staining compared to total surface area for each mouse. Data pooled from at least 3 experiments, *n* = three mice per cohort per experiment. Data shown as means ± SEM. Analyzed by one-way ANOVA with Tukey’s post hoc.
